# Exploring the Properties of Micronized Natural Zeolitic Volcanic Tuff as Cosmetic Ingredient

**DOI:** 10.3390/ma15072405

**Published:** 2022-03-24

**Authors:** Emilia Neag, Zamfira Stupar, Anamaria Iulia Torok, Ionut Surupaceanu, Marin Senila, Oana Cadar

**Affiliations:** 1 National Institute for Research and Development of Optoelectronics Bucharest INOE 2000, Research Institute for Analytical Instrumentation, 67 Donath Street, 400293 Cluj-Napoca, Romania; zamfira.dinca@icia.ro (Z.S.); iulia.torok@icia.ro (A.I.T.); marin.senila@icia.ro (M.S.); oana.cadar@icia.ro (O.C.); 2 Apel Laser SRL, Mogosoaia, 25 Vanatorilor Street, 077135 Ilfov, Romania; ionut.surupaceanu@apellaser.ro

**Keywords:** cosmetics, zeolites, micronization, stability tests, zeolitic volcanic tuff

## Abstract

This study explores the characteristics of a micronized natural zeolitic volcanic tuff (MZ) as ingredient in cosmetic formulations. In particular, the purpose was to prepare and investigate the organoleptic and physicochemical properties of two representative cosmetic formulations containing MZ. The MZ samples were characterized using X-ray fluorescence (XRF), X-ray diffraction (XRD) and Fourier transform infrared (FT-IR) spectroscopy. pH, cation exchange capacity (CEC), apparent density, chemical composition and particle size distribution of MZ samples were also determined. The micronization treatment applied did not produce significant structural and physicochemical changes with respect to the raw zeolitic volcanic tuff. The prepared formulations containing 5% MZ with different particle sizes (100–125 µm and 125–250 µm) were subjected to preliminary and accelerated stability tests, and the pH and organoleptic properties were also evaluated. The cosmetic formulations presented a pH of 4.3, a pleasant touch, good spreadability, easy application on skin, no color alteration and a good stability after 15, 30 and 60 days of storage at room temperature, low temperature and freezer during the accelerated stability tests. The obtained results endorse the MZ as suitable for the development of formulations exploiting the clinoptilolite properties as a cosmetic ingredient.

## 1. Introduction

Zeolites are crystalline, hydrated aluminosilicates of the alkali or alkaline earth metals, possessing unique and remarkable physicochemical properties [1,2]. These characteristics make them useful in industrial, agricultural, medical and health care applications [3,4,5]. Among the most abundant natural zeolites, clinoptilolite is capable of reducing the toxic effect of substances in the human body [4]. EFSA concluded, in 2013, that 10,000 mg clinoptilolite/kg complete feed can be safe for animals [4,6]. However, an adequate characterization of the zeolite material should be performed before its use in various products (often zeolitized rock has been considered to be zeolite, without taking into account the presence of other phases). Moreover, the minerals associated with natural zeolite could interfere, develop side effects or simply reduce the activity of zeolite [7].

The tribomechanical micronization process implies the reduction in the solid materials particles [8,9]. During the micronization activation process, the ion exchange capacity and the specific surface of the zeolites were improved [10]. The particle size distribution, the specific surface, the particle diameter and shape, the chemical and mineralogical composition, volume mass, surface activity and optical properties are the basic parameters of the micronizing product quality. The quality of micronized product depends on the physical, mechanical, chemical and mineralogical characteristics of the raw material [11]. Studies showed that the tribomechanical micronization of zeolites can have positive effects on various pathological conditions of organisms [8]. Moreover, the tribomechanical micronized clinoptilolite can be useful in healing of decubitus ulcer and psoriasis [12] and dyslipidemia treatment [13].

Over time, the demand for cosmetics products has increased throughout the world. During the manufacturing of cosmetic products (i.e., toothpastes, face make-up, lipsticks, etc.) the use of different mineral pigments leads to the contamination of the final product with trace amounts of As, Cd, Co, Cr, Cu, Pb, Hg, etc. [14,15,16]. High levels of metals were reported in cosmetic products, such as Pb in lipsticks (3.76 g/kg) and body care products (790 g/kg), Hg in skin-lightening creams (65.13 g/kg), Cd in kohl (6.26 g/kg), Al (50.0 g/kg), Ni (359.44 mg/kg) and As (11.1 mg As/kg) in eyeshadows. Unfortunately, only in some countries the content of metals in cosmetic products is prohibited or restricted by regulations, while in many countries no regulations are applied [14]. The regular application of cosmetic products containing toxic metals can cause acute health effects on human skin. Thus, it is recommended to measure the metal content in the raw materials used as ingredients in cosmetic formulations and also in the final product [15]. European Regulation No. 1223/2009 [17] establishes the requirements for cosmetic products available on the market and the restrictions for certain substances, which can cause adverse health effects to ensure the protection of the human health [18].

It is necessary to conduct stability studies to evaluate the physical integrity and safety of the cosmetic formulation for consumers [19]. The exposure of cosmetic products to various factors, such as temperature, light, humidity and packaging material can influence their stability, organoleptic and physico-chemical properties [20]. Very little research has been reported on the use of natural zeolites in the cosmetic industry [21]. Pesando et al. evaluated the ability of zeolite to retain Cd, Pb, Cr, Ni and Co in a new skin care formulation containing 1% to 3% zeolite. Their findings revealed the selectivity of zeolite for Cd and Ni adsorption in a 3% zeolite-based formulation [21].

The purpose of this study was to prepare and explore the properties of the micronized natural zeolitic volcanic tuff (MZ) in order to be used as ingredient in exfoliating masks. In this regard, the prepared MZ was characterized in terms of structural and physicochemical characteristics, and evaluation of preliminary and accelerated stability studies of cosmetic formulations containing MZ.

## 2. Materials and Methods

### 2.1. Micronized Zeolite Preparation

**Figure 1:**
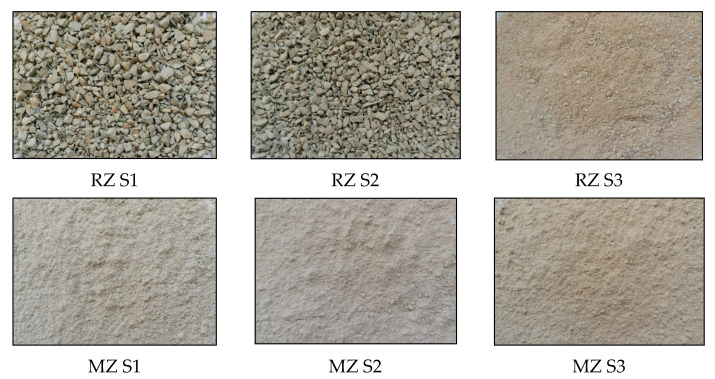
Zeolitic volcanic tuffs before (RZ S1, RZ S2 and RZ S3) and after micronization (MZ S1, MZ S2 and MZ S3).

Three samples (S1, S2 and S3) of natural zeolitic volcanic tuff denoted RZ (raw zeolitic volcanic tuff) were purchased from the Romanian market and considered as control samples. The RZ S1, RZ S2 and RZ S3 samples were grinded at a speed of 1500 rpm for 30 s using a vibratory disc mill (RS 200 Retsch) and micronized at a pressure of 12 bar using a micronization system (PilotMill-2 FPS1015, Como, Italy), denoted MZ S1, MZ S2 and MZ S3. The zeolitic volcanic tuffs before and after micronization are presented in Figure 1. The micronization treatment applied did not produce color changes with respect to the RZ samples.

### 2.2. Characterization

The pH of the RZ and MZ samples was determined in a 1:5 solid:water (*w/v*) suspension using a Seven Excellence multiparameter (Mettler Toledo, Greifensee, Switzerland) at 20 °C. The apparent density, expressed in g/mL, was determined by placing 5 g of sample into a 10 mL graduated cylinder and recording the volume. The apparent density, ρ (g/mL) was calculated as the ratio between the mass and the volume of the sample [22]. The cation exchange capacity (CEC) of RZ and MZ was determined using the sodium acetate method [23]. The amounts of exchangeable cations (Na^+^, K^+^, Ca^+^ and Mg^+^) replaced by NH_4_^+^ ions in the solution were determined using an inductively coupled plasma optical emission spectrometer (ICP-OES) Optima 5300 DV (Perkin Elmer, Waltham, MA, USA). The Si and Al concentrations were determined using a portable X-ray fluorescence analyzer, XRF (Bruker TRACER 5i, Karlsruhe, Germany) equipped with a Rh target X-ray tube operated at 50 kV with 4 W, while the Na, K, Ca Mg, Fe and Mn concentrations were measured using ICP-OES, after microwave-assisted acid digestion protocol using a microwave Xpert system (Berghof, Eningen, Germany) as previously reported [24]. The conversion to the corresponding oxide was performed by multiplying the element concentration with the appropriate factor [25]. The Ni, Cr, Co, Cu, Zn, Cd, Pb concentrations in RZ and MZ samples were also determined using ICP-OES. The Hg content in the samples was determined using an Automated Direct Hg Analyzer Hydra-C (Teledyne Instruments, Leeman Labs, Mason, OH, USA), based on thermal desorption atomic absorption spectrometry [24].

The FT-IR spectra of RZ and MZ samples were recorded on 1% KBr pellets in the range from 4000 to 400 cm^−1^, with a spectral resolution of 2 cm^−1^, using a BX II Fourier transform infrared spectrophotometer (Perkin Elmer, Waltham, MA, USA). Total surface area and pore radius were obtained from N_2_ adsorption–desorption isotherms using the Brunauer–Emmett–Teller (BET) method for total surface area evaluation and Dollimore–Heal model for porosity data. The isotherms were obtained using a Sorptomatic 1990 apparatus (Thermo Electron Corporation, Waltham, MA, USA). The X-ray diffraction (XRD) patterns of RZ and MZ samples were recorded at room temperature using a D8 Advance (Bruker, Karlsruhe, Germany) diffractometer with CuKα radiation (λ = 1.54060 Å), operating at 40 kV and 35 mA. The degree of crystallinity was calculated as the ratio between the area of all diffraction peaks and the total area of diffraction peaks and amorphous halo [25]. Afterward, the quantitative amount of amorphous phase was calculated by difference, using degree of crystallinity [26].

The particle size distribution of RZ and MZ samples was determined by sieve analysis. All the samples were dried at 105 °C for 8 h in a laboratory oven and left to cool down in a desiccator prior to analysis. A 50 g sample was sieved through the following stack of sieves: >2000 µm, 1000–2000 µm, 500–1000 µm, 250–500 µm, 125–250 µm, 100–125 µm, 63–100 µm, 43–63 µm and 20–43 µm using a Retsch Vibratory Sieve Shaker AS 200 control (Haan, Germany). The sieves were stacked with the largest aperture size at the top, and the smallest aperture at the bottom. The mass retained on each sieve was weighted and the percentage of each fraction was calculated considering the total mass of the sample.

### 2.3. Stability Evaluation of Cosmetic Formulations Containing MZ

#### 2.3.1. Composition of Cosmetic Formulations Containing MZ

The MZ samples were added into a cosmetic base purchased from the Romanian market. The ingredients of the cosmetic base were: aqua, Cocos nucifera (coconut) oil*, Helianthus annuus (sunflower) seed oil*, cetearyl alcohol, coco-glucoside, Butyrospermum parkii (Shea) butter*, phenoxyethanol, xanthan gum, Prunus Armeniaca (apricot) kernel oil, Aloe Barbadensis (aloe vera) leaf juice powder* (*organic ingredients), citric acid, tocopherol, lactic acid, benzoic acid and dehydroacetic acid. Eighteen cosmetic formulations containing 5% MZ of particle sizes in the range of 20–43 µm, 43–63 µm, 63–100 µm, 100–125 µm, 125–250 µm and 250–500 µm were prepared and evaluated for their stability in different conditions. The prepared formulations were stored in glass vials with a capacity of 50 g, which were then closed and allowed to stand for 24 h for formulation stability.

#### 2.3.2. Preliminary Stability Study

The cosmetic formulations containing MZ (triplicate) were subjected to: (i) centrifugation test, C (5 g of each cosmetic formulation was placed into centrifuge tubes and the tubes were centrifuged at 1000, 2000 and 3000 rpm for 30 min); (ii) thermal stress test, TS (5 g of each cosmetic formulation was placed in a water bath at temperatures of 40, 60, 80 °C for 30 min; when room temperature had been reached the organoleptic properties of the cosmetic formulations were determined) and (iii) light test, L (10 g of each cosmetic formulation was placed under artificial illumination using cool white fluorescent tubes at an intensity of 1200 lux, 16.21 µmol m^−2^ s^−1^, 16/8 h day/night cycle for 15 days at 20 ± 2 °C). The cosmetic formulations were examined for phase separation and changes in organoleptic characteristics.

#### 2.3.3. Accelerated Stability Study

The best formulations selected from the preliminary stability study were subjected to the following conditions: (i) low temperature, LT (2 g of each cosmetic formulation was stored at 5 ± 1 °C, the analysis were performed after 15, 30 and 60 days); (ii) freezer, F (2 g of each cosmetic formulation was stored at −20 ± 1 °C, the analysis were performed after 15, 30 and 60 days), (iii) room temperature, RT (2 g of each cosmetic formulation was stored at 20 ± 2 °C, protected from light, the analysis were performed after 15, 30 and 60 days), (iv) freezing/defrosting cycles, F/D (2 g of each cosmetic formulation was subjected to a freezing cycle at −20 ± 1 °C for 24 h, followed by a defrosting cycle at 20 ± 2 °C for 24 h, the analysis were performed after six freezing/defrosting cycles); (v) oven, O (2 g of each cosmetic formulation was stored at 45 ± 0.3 °C, the analysis were performed after 15 days).

#### 2.3.4. Acceptance Criteria

The tests were performed to verify if some modifications in terms of phase separation and changes in physical properties would occur. The cosmetic formulations evaluated in extreme conditions were compared to the control formulations. Slight changes for aspect were accepted for the cosmetic formulations kept at extreme conditions. pH variations smaller than 10% were considered acceptable.

#### 2.3.5. Analysis of Cosmetic Formulations

In order to establish the applicability of the MZ in the cosmetic industry, the organoleptic characteristics (aspect, color, odor, application touch), pH and performance tests of cosmetic formulation containing MZ were evaluated. The organoleptic characteristics were classified as follows: (i) for aspect, color and odor: normal (N); slightly modified (M) and intensely modified (IM); (ii) for application touch: easy application, pleasant touch (A); sticky, unpleasant touch (S) and very sticky, very unpleasant touch (V) [27].

The pH of the cosmetic formulations was determined using pH test strips. The pH test strips were inserted into the samples and maintained for 1 min. The color of the pH strip was compared to the color on the chart and the observed value was noted.

The performance test was performed as follows: 0.3 g of sample was spread on a glass slide (6.0 × 3 cm), to mimic the film formation after application to the skin. Afterwards, the sample was placed in an oven at 36.5 ± 0.3 °C and monitored every 5 min, until the drying process was completed [27]. Each experiment was carried out in triplicate.

A closed-vessel Xpert microwave system was used for sample digestion. An amount of 200 mg cosmetic formulation sample was digested using 5 mL HNO_3_ 65% and 2 mL H_2_O_2_ 30% in polytetrafluoroethylene digestion vessels, using a four-step digestion program (120 °C and 190 °C—heating; 100 °C and 25 °C—cooling) for a total digestion time of 35 min. When the vessels cooled down, the digested samples were transferred in volumetric flasks and diluted to the mark with ultrapure water. Each sample was prepared in triplicate. The resulting solutions were analyzed using an inductively coupled plasma quadrupole mass spectrometer, ICP-MS (ELAN DRC II, Perkin–Elmer, Waltham, MA, USA).

## 3. Results and Discussion

### 3.1. RZ and MZ Characteristics

In Table 1 are reported the obtained pH values for the RZ and MZ samples. The RZ samples showed high pH values, between 9.32 and 9.79, while the MZ samples showed slightly lower pH values (between 9.11 and 9.61) compared to RZ samples. The highest pH value found was for MZ S1 and the lowest value found was for MZ S2. The apparent density values obtained for the analyzed samples are given in Table 1. The RZ samples showed apparent density values in the range of 0.8 and 1.0 g/mL, while the MZ samples exhibited a slightly higher value of 1.1 g/mL than the RZ samples. The CEC values (Table 1) of the RZ samples (1.18–1.48 meq/q) were slightly lower than those of MZ samples (1.47–1.60 meq/q).

The highest CEC value found was for MZ S1, then for MZ S2, and the lowest value found was for MZ S3. No major differences in respect of pH, apparent density and CEC values were observed in the analyzed MZ samples compared to the RZ samples.

Low variations in the chemical composition of MZ compared to the RZ samples were observed (Table 1). Analyzing the obtained results, the major oxides found in the analyzed samples were SiO_2_ and Al_2_O_3_, while the other oxides (K_2_O, CaO, Na_2_O, MnO and MgO) were present in low amounts. The Al_2_O_3_ content in the MZ samples varied in the range 10.79–11.98%, while SiO_2_ content varied in the range 62.66–65.30%. The K_2_O, CaO and Na_2_O contents in the MZ samples varied in the ranges 1.31–2.59%, 3.47–4.45% and 0.72–1.66%, respectively. Small contents of MnO (0.02–0.09%) and MgO (0.54–0.94%) were found in the analyzed samples. The Si/Al ratio in MZ samples ranged between 4.61 and 5.34, while the Si/Al ratio for RZ ranged between 4.57 and 5.06. The Si/Al ratio of MZ S1 and MZ S2 samples slowly increased compared with RZ samples, except for the MZ S3 sample, whose Si/Al ratio decreased. A Si/Al ≥ 4.0 is specific for clinoptilolite-type zeolite. Generally, the Si/Al ratio is theoretically related to the CEC value. Zeolites with lower Si/Al ratio have higher CEC values [28]. As it can be seen, the Si/Al of MZ S1 was 5.20 and its CEC value was 1.60 meq/q, while the Si/Al and CEC value of RZ S1 was 4.89 and 1.18 meq/q, respectively.

#### 3.1.1. Trace Elements (Ni, Cr, Co, Cu, Zn, Cd, Pb and Hg) Concentrations

The differences in trace elements composition among the studied MZ samples can be observed in Table 2. The lowest Ni, Cr, Co, Cu concentrations were determined in the MZ S1, while the highest Ni, Cr, Co, Cu and Pb concentrations were found in the MZ S3 sample. As it can be observed, Cu content decreased in the MZ S1 and MZ S2 samples and slightly increased in the MZ S3 sample compared to the raw samples. A similar trend was observed in the case of Zn. In all cases, Cd and Hg concentrations were below the detection limit. The Ni concentration in the MZ S1 and MZ S2 samples was 2.6 mg/kg and 3.6 mg/kg, while in the MZ S3 sample was higher 10.7 mg/kg. The contents of Cr and Pb were higher in the MZ S3 sample compared to the MZ S1 and MZ S2 sample.

#### 3.1.2. Structure of RZ and MZ Samples

**Figure 2:**
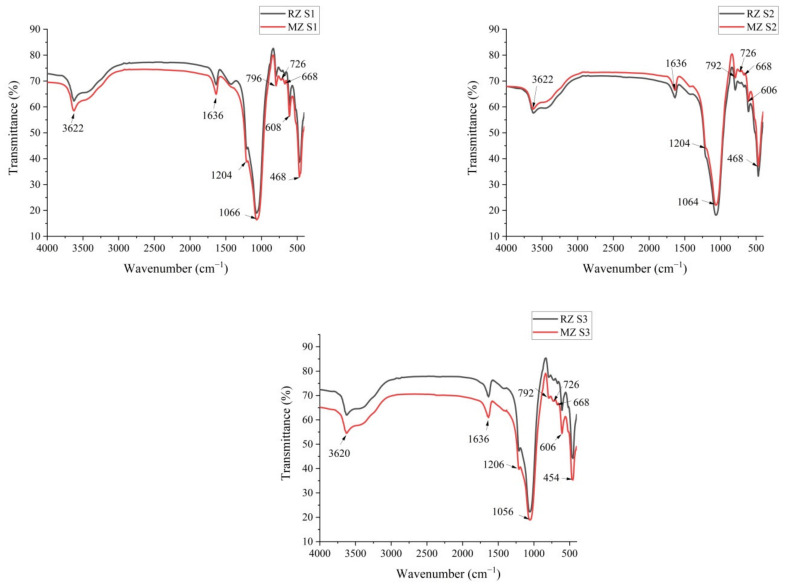
FT-IR spectra of RZ and MZ samples.

The FT-IR spectra of the samples before and after micronization are given in Figure 2. Analyzing the FT-IR spectra, the following bands were identified: the bands assigned to the presence of water in the zeolite structure (3700–1600 cm^−1^) and the bands attributed to the Si–O–Si and Si–O–Al vibrations in the region 1200–400 cm^−1^, namely, asymmetric stretching (1250–950 cm^−1^), symmetric stretching (720–650 cm^−1^) and T-O bending (500–400 cm^−1^), where T indicates the tetrahedral position of Si or Al [29,30].

The band at 3620 cm^−1^ represents the stretching vibrations of O-H, while the bands 1636 and 1634 cm^−1^ are attributed to the bending vibrations of H-O-H [29]. Previous studies showed that zeolites adsorbed water molecules. In particular, natural and synthetic zeolites were tested to separate the H_2_O/H_2_SO_4_ and H_2_O/C_2_H_5_OH mixtures. The results showed that the zeolite Type 3A and Heulandite adsorbed very rapidly the water molecules with increasing temperature [31]. Generally, the clinoptilolite bands are in the region 1630 cm^−1^ to 1650 cm^−1^ [30]. The absorption bands at 1204, 1202 and 1206 cm^−1^ correspond to the internal asymmetric vibrations of the T-O bonds, while the bands at 1056 and 1064 cm^−1^ are assigned to external asymmetric T-O stretching vibration. The low intensity bands at 796, 792, 726 and 668 cm^−1^ are ascribed to symmetrical T-O stretching vibrations. The bands 606 and 608 cm^−1^ can be attributed to the external vibrations of T-O tetrahedral units coupled in rings [29]. Finally, the bands at 454 and 468 cm^−1^ correspond to the bending vibrations of Si-O [29,32,33]. Arcoya et al. reported that after calcination at 973 K (700 °C) of a natural clinoptilolite a decrease in the intensity of the characteristic peaks of the zeolite was observed and at 1073 K (800 °C) the collapse of the structure was complete [34]. Akkoca et al. reported the collapse at 550 °C of zeolites belonging to earth alkali clinoptilolite with low alkali content [35].

Yokoi stated that zeolites, as porous crystalline materials, have a uniform pore size of 0.3–1 nm. MZ S3 sample has a BET surface area of 33 m^2^/g and a pore volume of 0.13 cm^3^/g [36].

**Figure 3:**
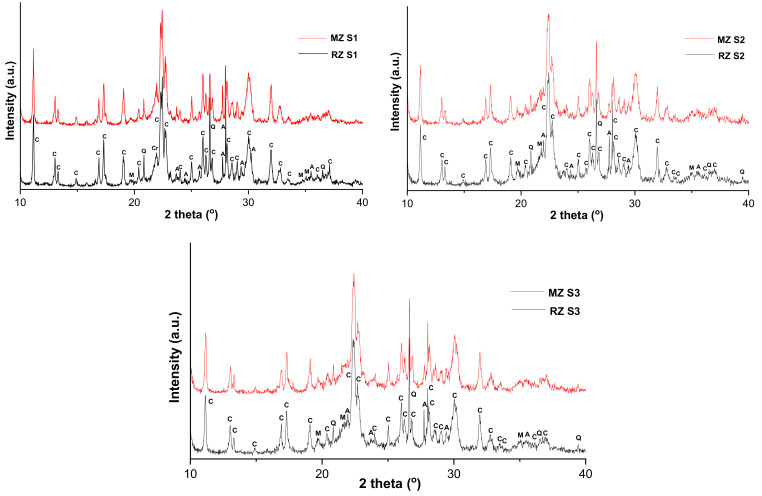
The XRD patterns of RZ and MZ samples (note: clinoptilolite, C; albite, A; muscovite, M; quartz, Q; cristobalite, Cr).

Since the mineral formation depends mainly on the geological site and physicochemical conditions, the zeolite deposits generally embody a heterogeneous mixture of zeolite minerals together with several gangue minerals, such as quartz, feldspars and phyllosilicates (micas, clay minerals) [37]. Generally, the XRD patterns of investigated RZ and MZ volcanic tuffs display similar crystalline phases, but different crystal structure (Figure 3). In this regard, the XRD patterns of RZ S1 and MZ S1/ RZ S2 and S3, and MZ S2 and S3 volcanic tuffs indicates the presence of clinoptilolite (PDF 00-0147-1870/ PDF 00-039-1383) as main phase, accompanied by muscovite (PDF 01-089-7539/ PDF 00-058-2034), albite (PDF 00-010-0393/ PDF 00-041-1480) and quartz (PDF 01-070-7344/ PDF 00-046-1045). In case of the RZ S1 and MZ S1 samples, the presence of cristobalite (PDF 01-074-9378) is also remarked. The XRD patterns of the RZ and MZ volcanic tuffs display the typical diffraction peaks of clinoptilolite zeolite structure (2θ around 11, 22, 26, 30 and 32°) [25]. Clinoptilolite is the most abundant natural zeolite and is widely used in many applications [37]. The RIR (Reference Intensity Ratio) method [38] used for the quantitative phase analysis, indicates that the RZ S1-3 and MZ S1-3 volcanic tuffs are composed mainly of clinoptilolite-type zeolite (77/70/71%) accompanied by plagioclase feldspars (6/8/9%), silica polymorphs (9/4/5%) and clay minerals (4/18/16%). Moreover, it can be noticed that after micronization, the zeolite mineral content has not decreased compared to the initial sample, the effects of amorphization due to the micronization being poorly expressed. The non-crystalline components were not quantified by the XRD analysis, but the presence of amorphous volcanic glass in RZ and MZ volcanic tuffs is indicated by the broad diffraction hump centered at 2θ ≈ 25°. The degree of crystallinity of studied samples was similar before and after micronization, namely, 76% (RZ and MZ-S1), 68% (RZ and MZ-S2) and 67% (RZ and MZ-S3), respectively. The low level of amorphous phase content in RZ and MZ samples could be mainly attributed to the presence of quartz and kaolinized volcanic ash [26].

#### 3.1.3. Particle Size Distribution

The results of the particle size distribution of RZ and MZ samples are presented in Table 3. After micronization, a noticeable change in the particle size distribution of the samples was observed. During micronization, the zeolite particles were broken in smaller fragments causing changes in the particle size distribution. Thus, the percentage of the particles from the >2000 µm and 1000–2000 µm class began (RZ samples) to decrease considerably, while the content of the finest particles began to increase (MZ samples). After micronization, the percentage of the 20–43 µm, 43–63 µm and 63–100 µm classes increased from 22.18% to 56.74%, 9.41% to 25.58% and 9.96% to 18.77%, respectively. The percentage of the 100–125 µm, 125–250 µm and 250–500 µm class increased from 3.72% to 4.80 %, 14.58% to 15.19% and 2.97% to 11.68%, respectively.

### 3.2. Stability of Cosmetic Formulations Containing MZ

#### 3.2.1. Analysis of Cosmetic Formulations

The cosmetic formulations containing three samples of MZ (5%) of different particle sizes were evaluated to determine their pH, drying time and trace element concentrations. The obtained results are presented in Table 4.

The pH of the prepared cosmetic formulations containing MZ of different particle sizes ranged from 4.2 to 4.4. The pH of the skin ranges from 4 to 6 [18]. A slight increase in pH with the decrease in MZ particle size was observed, namely the pH of MZ S1 5% 20–43 µm was 4.4, while the pH of MZ S1 5% 250–500 µm was 4.2. A decrease in drying time with the increase in MZ particle size up to 125–250 µm was observed for all the studied samples, namely for MZ S1 the drying time decreased from 34.0 min (MZ S1 5% 20–43 µm) to 27.0 min (MZ S1 5% 125–250 µm), for MZ S2 the drying time decreased from 33.4 min (MZ S2 5% 20–43 µm) to 32.3 min (MZ S2 5% 125–250 µm), for MZ S3 the drying time decreased from 35.6 min (MZ S3 5% 20–43 µm) to 30.0 min (MZ S3 5% 125–250 µm). The highest drying time was observed for the cosmetic formulation containing MZ of 250–500 µm particle size, while the lowest drying time was observed for the cosmetic formulation containing MZ of 125–250 µm particle size, among the studied zeolite samples.

The Cr concentration in the cosmetic formulations, from 0.17 to 2.59 mg/kg, was lower than the maximum limit of 50 mg/kg set by USFDA [15]. The highest Cu concentration was 0.74 mg/kg for MZ S2 5% 250–500 µm, while the lowest concentration was 0.15 mg/kg for MZ S1 5% 43–63 µm. The Ni concentration varied from 0.46 to 2.28 mg/kg, below the recommended level of 200 mg/kg established by USFDA [15]. Pb concentrations were below the limit established by USFDA (20 mg/kg) [15] in all cosmetic formulations. The lowest Pb concentration was found in MZ S1 5% 250–500 µm (0.93 mg/kg). It can be noted that the concentration of Pb decreases as the particle size of MZ increases. MZ S1 5% 43–63 µm contains the highest Zn concentration (3.92 mg/kg), while MZ S2 5% 100–125 µm contains the lowest Zn concentration (0.68 mg/kg). The As, Cd and Co concentrations were below detection limit (0.10 mg/kg) in all the cosmetic formulations.

#### 3.2.2. Preliminary Stability Study

Based on the results obtained (Table 5), the combinations chosen for preliminary and accelerated stability tests were MZ S1 5% 100–125 µm and MZ S1 5% 125–250 µm.

No relevant changes occurred regarding pH during the C and TS tests performed compared to the control formulations. After L test, the pH of the cosmetic formulations slightly increased up to 4.6 (cosmetic formulations became slightly more fluid). The formulations presented a slight liquid consistency after TS test at temperature of 60 and 80 °C and phase separation after centrifugation (the white cosmetic base at the top and zeolite at the bottom). No change of color occurred for both formulations during the tests. The cosmetic formulations presented a characteristic odor, without changes during the tests performed, and also a pleasant touch, good spreadability and easy application on skin. The modifications observed for aspect in the TS, C and L tests and for pH in the L test (pH variations were smaller than 10%) were accepted, due to the drastic conditions used to test the stability of the formulations.

#### 3.2.3. Accelerated Stability Study

Organoleptic characteristics and pH values of the cosmetic formulations in the accelerated stability study are presented in Table 6.

Generally, the organoleptic properties of both cosmetic formulations remained unchanged after 15, 30 and 60 days at LT, F and RT conditions. Modifications of aspect were not observed at LT, F and RT conditions in any of the cosmetic formulations. However, the aspect of the formulations changed after six F/D and O tests. The formulations had a slightly liquid consistency in both cases. These changes were expected since drastic conditions were used to test the stability of the formulations and accepted. Color alternations were not observed in the formulation subjected to LT, F, RT and F/D, indicating good stability. After O tests, the white color of the base was turned to beige. The pH values of cosmetic formulations remained stable during 15, 30 and 60 days at LT, F, RT and F/D, while the pH of cosmetic formulation increase up to 4.6 after O tests. No odor changes were observed during the tests performed in any of the cosmetic formulations. During the tests, the cosmetic formulations containing 5% MZ presented a pleasant touch, good spreadability and easy application on skin.

## 4. Conclusions

The preparation, characterization and evaluation of a micronized zeolitic volcanic tuff to verify its suitability as an ingredient in cosmetic formulations were investigated. No significant structural and compositional changes were detectable in respect to raw samples after micronization treatment. The cosmetic formulations containing 5% MZ of 100–125 µm and 125–250 µm particle sizes exhibited a good stability during the preliminary and accelerated stability tests. Moreover, the Cr, Ni, Pb concentrations were below the maximum limits established, while the As, Cd and Co concentrations were below detection limit of 0.10 mg/kg in both cosmetic formulations. No significant changes were observed in organoleptic properties and pH of the formulations during the storage. Moreover, the formulations were generally stable under stress conditions. The microbial stability and health risk assessment of the cosmetic formulations should be investigated further.

## Figures and Tables

**Table 1:** Physicochemical properties of RZ and MZ samples.

	pH	CEC	ρ	K_2_O	MnO	CaO	MgO	Fe_2_O_3_	SiO_2_	Al_2_O_3_	Na_2_O	Si/Al
		meq/q	g/mL	wt. %								
RZ S1	9.79	1.18	0.8	1.85	0.04	6.25	0.79	1.94	59.07	10.65	1.43	4.89
MZ S1	9.61	1.60	1.1	2.26	0.02	3.47	0.54	1.39	65.09	11.04	1.66	5.20
RZ S2	9.35	1.38	0.8	1.73	0.10	3.19	0.66	1.25	64.24	12.39	0.50	4.57
MZ S2	9.11	1.54	1.1	2.59	0.03	4.34	0.92	1.48	65.30	10.79	0.72	5.34
RZ S3	9.32	1.48	1.0	1.15	0.05	3.96	0.81	2.43	65.85	11.46	0.70	5.06
MZ S3	9.24	1.47	1.1	1.31	0.09	4.45	0.94	2.58	62.66	11.98	0.78	4.61

**Table 2:** Trace element concentrations of RZ and MZ samples.

Samples	Ni	Cr	Co	Cu	Zn	Cd	Pb	Hg
RZ S1	2.5	2.9	<1.0	27.9	64.1	<1.0	21.8	<0.005
MZ S1	2.6	4.1	<1.0	8.4	39.1	<1.0	8.7	<0.005
RZ S2	3.6	2.1	1.1	10.2	19.8	<1.0	28.5	<0.005
MZ S2	3.6	5.4	<1.0	9.2	15.3	<1.0	8.4	<0.005
RZ S3	2.1	<1.0	<1.0	7.9	14.0	<1.0	<1.0	<0.005
MZ S3	10.7	11.1	2.9	9.7	15.5	<1.0	18.4	<0.005

**Table 3:** Particles size distribution of RZ and MZ samples.

Samples	>2000	1000–2000	500–1000	250–500	125–250	100–125	63–100	43–63	20–43	0–20
	%									
RZ S1	58.90	30.40	7.10	1.40	<1.0	<1.0	<1.0	-	-	-
MZ S1	-	-	-	2.97	15.19	4.80	9.96	25.58	40.39	1.11
RZ S2	48.60	50.50	<1.0	-	-	-	-	-	-	-
MZ S2	-	-	-	3.43	15.12	3.72	10.42	9.41	56.74	1.15
RZ S3	<1.0	17.30	24.20	15.20	26.10	4.90	8.40	2.90	<1.0	-
MZ S3	-	2.67	12.47	11.68	14.58	4.65	18.77	13.01	22.18	-

**Table 4:** pH, drying time and trace element concentrations of cosmetic formulations containing 5% MZ of different particle sizes.

Formulations	pH	Drying Time	Cr	Cu	Ni	Pb	Zn
		min	mg/kg				
MZ S1 5% 20–43 µm	4.4	34.0	0.17	<0.10	1.40	18.08	1.80
MZ S1 5% 43–63 µm	4.3	33.0	<0.10	0.15	1.82	16.35	3.92
MZ S1 5% 63–100 µm	4.2	32.5	2.11	0.39	1.84	2.58	2.07
MZ S1 5% 100–125 µm	4.2	31.4	0.98	0.21	1.30	2.04	1.09
MZ S1 5% 125–250 µm	4.2	27.0	0.85	0.33	0.78	1.71	2.06
MZ S1 5% 250–500 µm	4.2	36.0	0.26	0.40	0.46	0.94	1.34
MZ S2 5% 20–43 µm	4.3	33.4	0.40	0.50	1.52	14.57	2.01
MZ S2 5% 43–63 µm	4.2	33.3	0.18	0.51	1.09	4.47	0.94
MZ S2 5% 63–100 µm	4.2	33.3	<0.10	0.18	2.28	2.04	1.10
MZ S2 5% 100–125 µm	4.2	33.1	0.95	0.27	0.68	1.86	0.68
MZ S2 5% 125–250 µm	4.2	32.3	1.89	0.47	1.55	2.05	1.89
MZ S2 5% 250–500 µm	4.3	35.0	0.51	0.74	0.69	1.26	1.65
MZ S3 5% 20–43 µm	4.4	35.6	2.59	0.54	1.63	16.13	1.75
MZ S3 5% 43–63 µm	4.3	35.4	0.47	0.42	0.85	2.25	3.54
MZ S3 5% 63–100 µm	4.3	35.0	1.32	0.39	0.71	3.96	1.03
MZ S3 5% 100–125 µm	4.2	32.0	0.18	0.41	0.92	1.48	1.09
MZ S3 5% 125–250 µm	4.2	30.0	0.19	0.38	1.53	1.78	3.00
MZ S3 1% 250–500 µm	4.2	36.0	0.50	0.36	1.05	0.93	0.74

**Table 5:** Organoleptic characteristics and pH values of the cosmetic formulations in the preliminary stability study.

	Test	Aspect	Color	Odor	Application Touch	pH
MZ S1 5% 100–125 µm	C 1000 rpm, 30 min	M	N	N	A	4.3
	C 2000 rpm, 30 min	M	N	N	A	4.3
	C 3000 rpm, 30 min	M	N	N	A	4.3
	TS 40 °C, 30 min	N	N	N	A	4.2
	TS 60 °C, 30 min	M	N	N	A	4.2
	TS 80 °C, 30 min	M	N	N	A	4.3
	L	M	N	N	A	4.6
MZ S1 5% 125–250 µm	C 1000 rpm/30 min	M	N	N	A	4.3
	C 2000 rpm/30 min	M	N	N	A	4.3
	C 3000 rpm/30 min	M	N	N	A	4.3
	TS 40 °C, 30 min	N	N	N	A	4.3
	TS 60 °C, 30 min	M	N	N	A	4.3
	TS 80 °C, 30 min	M	N	N	A	4.3
	L	M	N	N	A	4.6

C—centrifugation; TS—thermal stress; L—light test; M—slightly modified; N—normal; A—easy application, pleasant touch.

**Table 6:** Organoleptic characteristics and pH values of the cosmetic formulations in the accelerated stability study.

	Test	Aspect	Color	Odor	Application Touch	pH
MZ S1 5% 100–125 µm	LT day 15	N	N	N	A	4.3
	LT day 30	N	N	N	A	4.3
	LT day 60	N	N	N	A	4.3
	F day 15	N	N	N	A	4.3
	F day 30	N	N	N	A	4.3
	F day 60	N	N	N	A	4.3
	RT day 15	N	N	N	A	4.3
	RT day 30	N	N	N	A	4.3
	RT day 60	N	N	N	A	4.3
	F/D	M	N	N	A	4.3
	O 45 °C	M	M	N	A	4.6
MZ S1 5% 125–250 µm	LT day 15	N	N	N	A	4.3
	LT day 30	N	N	N	A	4.3
	LT day 60	N	N	N	A	4.3
	F day 15	N	N	N	A	4.3
	F day 30	N	N	N	A	4.3
	F day 60	N	N	N	A	4.3
	RT day 15	N	N	N	A	4.3
	RT day 30	N	N	N	A	4.3
	RT day 60	N	N	N	A	4.3
	F/D	M	N	N	A	4.3
	O 45 °C	M	M	N	A	4.6

LT—Low temperature; F—Freezer; RT—Room temperature; F/D—Freezing/defrosting cycles; O—oven; M—slightly modified; N—normal; A—easy application, pleasant touch.

## Data Availability

Not applicable.
